# Affecting *Pseudomonas aeruginosa* Phenotypic Plasticity by Quorum Sensing Dysregulation Hampers Pathogenicity in Murine Chronic Lung Infection

**DOI:** 10.1371/journal.pone.0112105

**Published:** 2014-11-24

**Authors:** Roslen Bondí, Marco Messina, Ida De Fino, Alessandra Bragonzi, Giordano Rampioni, Livia Leoni

**Affiliations:** 1 Dept. of Sciences, University Roma Tre, Rome, Italy; 2 Infections and Cystic Fibrosis Unit, Division of Immunology, Transplantation and Infectious Diseases, IRCCS - San Raffaele Scientific Institute, Milan, Italy; 3 Italian Cystic Fibrosis Research Foundation c/o Ospedale Maggiore, Verona, Italy; Ghent University, Belgium

## Abstract

In *Pseudomonas aeruginosa* quorum sensing (QS) activates the production of virulence factors, playing a critical role in pathogenesis. Multiple negative regulators modulate the timing and the extent of the QS response either in the pre-quorum or post-quorum phases of growth. This regulation likely increases *P. aeruginosa* phenotypic plasticity and population fitness, facilitating colonization of challenging environments such as higher organisms. Accordingly, in addition to the factors required for QS signals synthesis and response, also QS regulators have been proposed as targets for anti-virulence therapies. However, while it is known that *P. aeruginosa* mutants impaired in QS are attenuated in their pathogenic potential, the effect of mutations causing a dysregulated timing and/or magnitude of the QS response has been poorly investigated so far in animal models of infection. In order to investigate the impact of QS dysregulation on *P. aeruginosa* pathogenesis in a murine model of lung infection, the QteE and RsaL proteins have been selected as representatives of negative regulators controlling *P. aeruginosa* QS in the pre- and post-quorum periods, respectively. Results showed that the *qteE* mutation does not affect *P. aeruginosa* lethality and ability to establish chronic infection in mice, despite causing a premature QS response and enhanced virulence factors production in test tube cultures compared to the wild type. Conversely, the post-quorum dysregulation caused by the *rsaL* mutation hampers the establishment of *P. aeruginosa* chronic lung infection in mice without affecting the mortality rate. On the whole, this study contributes to a better understanding of the impact of QS regulation on *P. aeruginosa* phenotypic plasticity during the infection process. Possible fallouts of these findings in the anti-virulence therapy field are also discussed.

## Introduction

Quorum sensing (QS) is an intercellular communication process based on the synthesis and secretion of signal molecules that bind to cognate receptors. The signal-activated receptors trigger the expression of target genes. Since the concentration of signal molecules is proportional to cell density, QS coordinates gene expression when the bacterial population reaches a critical threshold level. The population density at which gene expression is triggered is called the “quorum”, while the phase before expression is called the “pre-quorum” period [1]–[3].

QS processes are widespread in the bacterial world and they are studied with particular intensity in *Pseudomonas aeruginosa*. This bacterium is one of the most dreaded Gram-negative pathogens in developed countries, being responsible for both community- and hospital-acquired infections. In addition, *P. aeruginosa* chronic lung infection is the major cause of death in patients with cystic fibrosis (CF), a genetic disease affecting about 1/3,000 newborns in the Caucasian population. *P. aeruginosa* infections are difficult to eradicate as a consequence of intrinsic antibiotic resistance and growth in bacterial communities referred to as biofilms [4], [5]. Since in *P. aeruginosa* QS plays a critical role in the production of virulence factors and in biofilm formation, it is considered a very promising target for the development of anti-virulence drugs [6]–[9].

**Figure 1:**
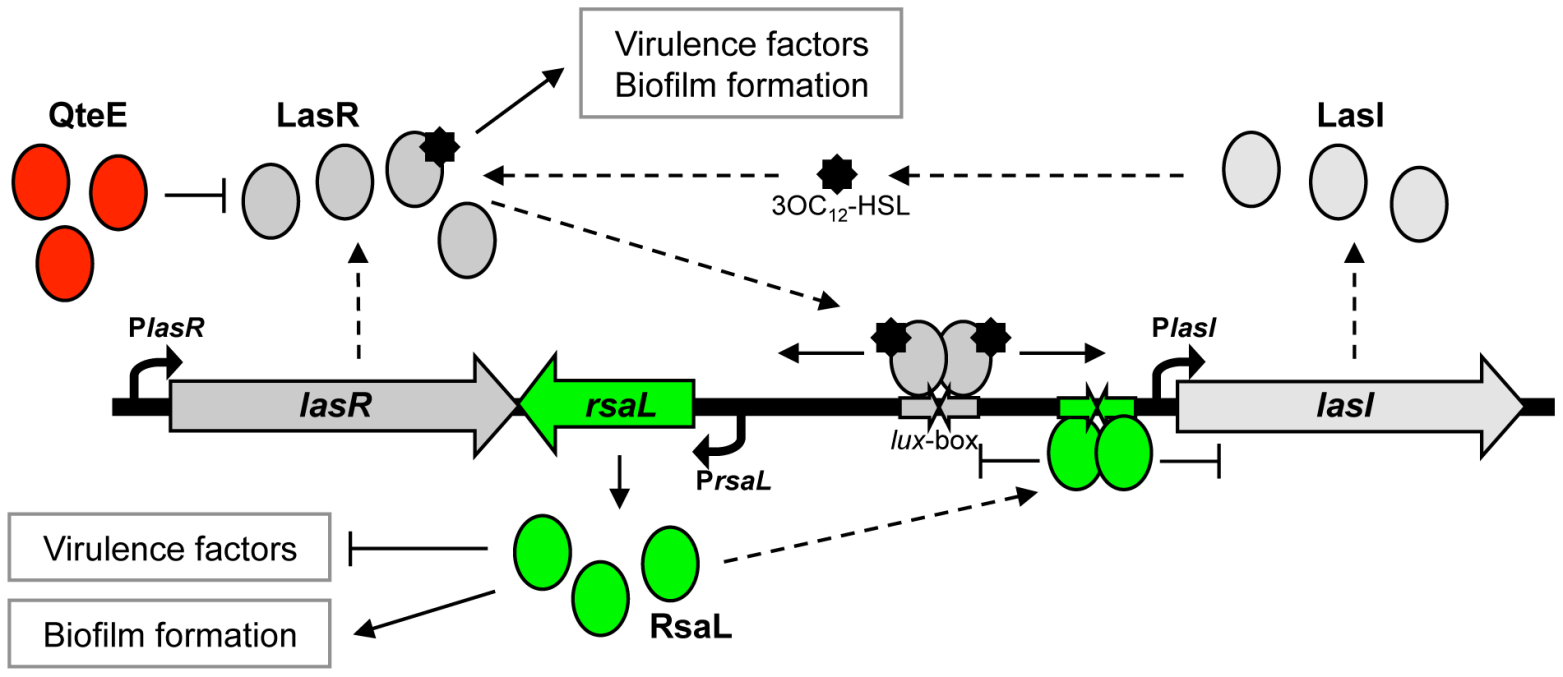
Schematic representation of QteE- and RsaL-dependent regulation of the *P. aeruginosa las* QS system. In the pre-quorum period, QteE binds to the LasR receptor and prevents the binding of the LasR-3OC_12_-HSL complex to the *rsaL*-*lasI* bidirectional promoter [20], hence delaying the onset of the QS response. Once the quorum has been reached, the LasR/3OC_12_-HSL complex triggers the transcription of both *rsaL* and *lasI* genes. The consequent increase of 3OC_12_-HSL levels, and thus of activated LasR, generates a positive feedback loop also responsible for the increase of RsaL levels. RsaL binding to the *rsaL*-*lasI* bidirectional promoter represses the expression of both *rsaL* and *lasI* genes, thus counteracting the positive feedback loop. This circuit provides 3OC_12_-HSL homeostasis [24]. Solid arrows represent positive control; T-shaped lines represent negative control; dashed arrows indicate information flow; curved arrows represent the transcription start points of the indicated genes.

*P. aeruginosa* has at least three QS systems based on the production, secretion and perception of distinct signals: *N*-3-oxo-dodecanoyl-homoserine lactone (3OC_12_-HSL), *N*-butyryl-homoserine lactone (C_4_-HSL), and molecules belonging to the 2-alkyl-4-quinolones (AQs) family. The signal molecule 3OC_12_-HSL is required for optimal production of the other QS signals, though this hierarchy is dependent upon growth conditions [3], [10]–[13]. 3OC_12_-HSL is produced by the synthase LasI, encoded by the *lasI* gene, and perceived by the signal receptor LasR, encoded by *lasR* (Fig. 1). The LasR/3OC_12_-HSL complex activates the transcription of hundreds of genes, including: *i*) the *lasI* gene, thus generating the autoinduction circuit typical of most QS systems; *ii*) the genes required for C_4_-HSL and AQs synthesis and reception; *iii*) many genes involved in virulence factors production and biofilm formation [3], [10], [11].

In *P. aeruginosa* the autoinduction circuit is coupled to multiple negative regulatory mechanisms that modulate the timing and the extent of the QS response, hence limiting the metabolic burden for virulence factors production when unnecessary. Overall, this fine-tuning is believed to increase *P. aeruginosa* phenotypic plasticity and population fitness, ultimately facilitating colonization of challenging environments such as higher organisms' tissues [3], [14]–[17].

Multiple negative modulators delaying the onset of the QS response by restraining the expression of the *lasI* gene in the pre-quorum period have been identified so far [18]–[21]. As an example, QteE decreases LasR stability at low cell density. Accordingly, in a *qteE* mutant the transcription of both *lasI* and the 3OC_12_-HSL-dependent gene *lasB*, encoding the secreted virulence factor elastase, is prematurely activated (Fig. 1) [17], [20]. Moreover, the overexpression of *qteE* causes a strong reduction of virulence in potato and fruit fly models of infection [22], suggesting that QteE might limit *P. aeruginosa* virulence also in mammals. However, artificial gene overexpression could produce aberrant effects, and the impact of *qteE* mutation on 3OC_12_-HSL and virulence factor production, and on the overall pathogenic potential of *P. aeruginosa* in either test tube cultures or animal models of infections is still unexplored.

After the QS threshold has been reached, in the post-quorum growth phase, additional regulatory factors may intervene, modulating the overall levels of signal molecule produced by the bacterial population. The RsaL protein, a repressor of *lasI* transcription encoded by the *rsaL* gene, is the best characterized post-quorum regulator in *P. aeruginosa*. Since the LasR/3OC_12_-HSL complex activates both *rsaL* and *lasI* transcription, it generates a homeostatic regulatory loop that allows a population of *P. aeruginosa* cells to avoid 3OC_12_-HSL accumulation in the post-quorum period (Fig. 1) [23]–[25]. The effect of the *rsaL* mutation on 3OC_12_-HSL and virulence factor production in test tube cultures and in an insect infection model has been thoroughly investigated in a previous study [23], [26]. Briefly, it has been shown that *rsaL* mutation leads to enhanced motility and secreted virulence factors production such as pyocyanin and elastase, resulting in a hypervirulent phenotype in the *Galleria mellonella* model of systemic (acute) infection. On the other hand, the *rsaL* mutant strain displays increased sensitivity to different antibiotics and reduced ability to form biofilm [26].

*P. aeruginosa* mutants impaired in QS are less pathogenic in various animal models of infection, especially in those developed to investigate the acute infection process [27]. However, despite *P. aeruginosa* biofilm-based chronic infections have higher social and economic costs in developed countries [4], [28], studies on mammalian models of chronic infection have received less attention so far, likely due to the complexity to establish long-term chronic infection in animal models [27], [29], [30].

Given the importance of QS and phenotypic plasticity in *P. aeruginosa* pathogenicity, it is believed that pre- and post-quorum control of QS response could play a key role in the establishment and persistence of the infection [15], [16]. Accordingly, ancillary regulators controlling the timing and extent of the QS response have been proposed as targets for the development of anti-virulence therapies against *P. aeruginosa* [31]. However, to the best of our knowledge, the effect of mutations causing a dysregulated timing and/or magnitude of the QS response on *P. aeruginosa* virulence has never been investigated in mammalian models of chronic infection.

The general objective of this study has been to investigate the impact of QS dysregulation in *P. aeruginosa* pathogenesis, by selecting QteE and RsaL as representatives of two negative regulators that control the *las* QS system in the pre- and post-quorum periods, respectively. As summarized above, the effect of the *rsaL* mutation in test tube cultures has been thoroughly characterized by our group in previous studies, while the effect of this mutation in animal infection models has not been investigated so far [23], [24], [26]. On the other hand, very little is known about the effect of the *qteE* mutation on *P. aeruginosa* 3OC_12_-HSL production and virulence, both *in vitro* and *in vivo*. For this reason, the first part of this study has been focused on the characterization of the *qteE* mutant virulence-related phenotypes in test-tube cultures. Next, the effect of both *qteE* and *rsaL* mutations on *P. aeruginosa* pathogenesis has been investigated in a murine model of chronic infection. Results showed that a mutation in *qteE* causes a premature QS response and hyperproduction of virulence factors in *P. aeruginosa* cultures. However, the anticipation of the QS response in the pre-quorum period due to the *qteE* mutation does not affect *P. aeruginosa* pathogenicity, while the post-quorum dysregulation caused by the *rsaL* mutation hampers the establishment of chronic lung infection. Overall these findings contribute to fill-in the current gap of knowledge about the relevance of QS modulation in *P. aeruginosa* pathogenesis, and stimulate a re-discussion of the overall role played by QS during the infection process.

## Results and Discussion

### Phenotypic characterization of the *P. aeruginosa qteE* mutant

The effect of *qteE* mutation on *P. aeruginosa* 3OC_12_-HSL-dependent response was determined along growth by comparing the levels of this signal molecule and of selected QS-dependent virulence factors in wild type and in *qteE* cultures carrying either the empty vector pBBR1MCS-5 or its derivative plasmid (named pQteE) expressing the *qteE* gene.

**Figure 2:**
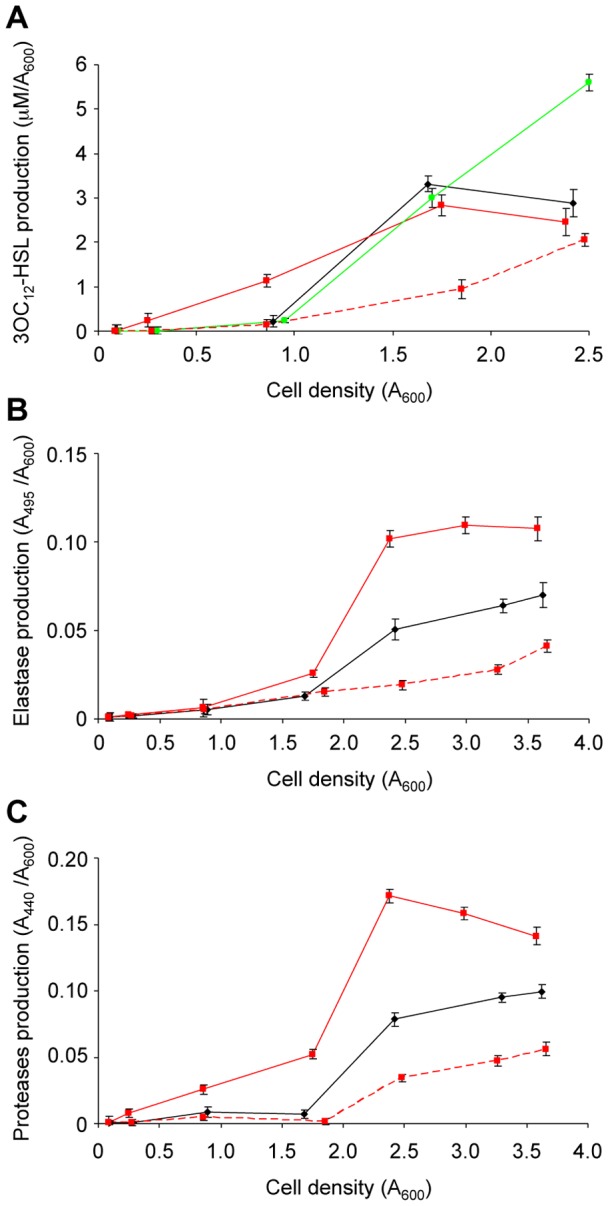
Effect of QS dysregulation caused by *qteE* mutation on *P. aeruginosa* virulence-related phenotypes. Levels of (A) 3OC_12_-HSL, (B) elastase, (C) proteases produced along growth by *P. aeruginosa* wild type (black lines), *qteE* (red lines) and *rsaL* strains (green line) carrying the pBBR1MCS-5 empty vector, or by the *qteE* strain carrying the pQteE plasmid (pBBR1MCS-5-derived) for the expression of *qteE* (dashed red line). Values are the means (± standard deviations) of at least three independent experiments.

As shown in Figure 2A, the *qteE* mutant produced detectable levels of 3OC_12_-HSL earlier than the wild type strain, reaching a 3OC_12_-HSL concentration about 6-fold higher at A_600_≈1. Interestingly, 3OC_12_-HSL levels measured in the *qteE* and in the wild type strains plateaued at the same level in the post-quorum phase of growth (A_600_≈2). This trend of 3OC_12_-HSL production in the *qteE* mutant is also consistent with previous western hybridization experiments showing that the positive effect of the *qteE* mutation on LasR protein stability is restricted to the pre-quorum period [20]. Conversely, as previously shown [24], the *rsaL* mutant disclosed normal 3OC_12_-HSL production in the pre-quorum period, while this mutant produced higher 3OC_12_-HSL levels than the wild type strain after the QS threshold has been reached (A_600_>1.8; Fig. 2A).

**Figure 3:**
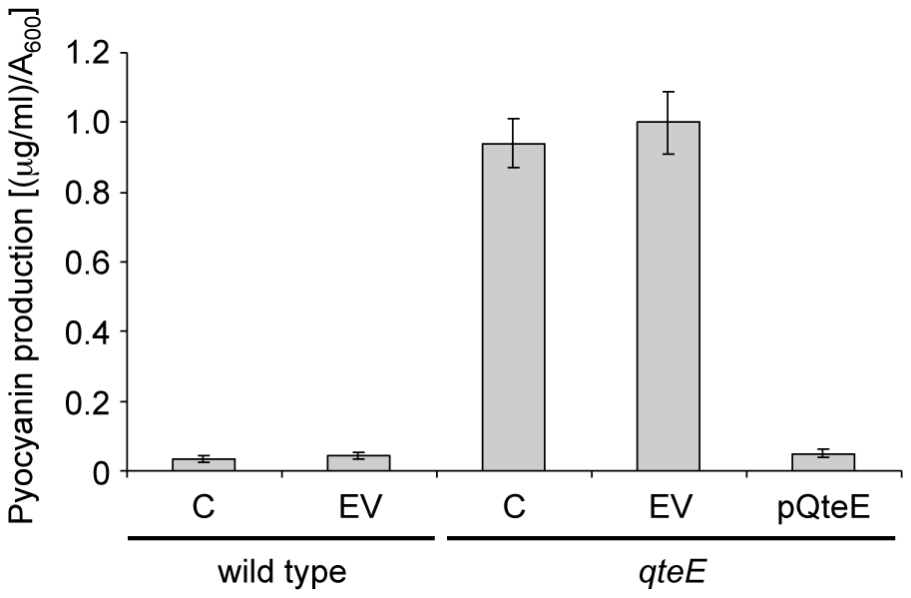
Effect of QS dysregulation caused by *qteE* mutation on pyocyanin production in *P. aeruginosa*. Levels of pyocyanin measured in cell-free supernatants from cultures of the indicated strains grown till A_600_≈3.5. C, no plasmid; EV, pBBR1MCS-5 empty vector; pQteE, pBBR1MCS-5 derivative plasmid for *qteE* expression.

In agreement with the precocious synthesis of 3OC_12_-HSL, the *qteE* mutant also anticipated the production of elastase (Fig. 2B) and protease (Fig. 2C). Differently from 3OC_12_-HSL levels, it seems that the anticipated expression of proteases and elastase levels in the *qteE* mutant causes accumulation of these secreted factors also in the post-quorum period (compare panels A, B and C of Fig. 2). The homeostatic control of 3OC_12_-HSL levels in the post-quorum period is likely due to specific mechanisms that do not affect proteases and elastase production, including the transcriptional repression exerted by RsaL on *lasI*, and the activity of the acyl-HSL degrading enzymes produced by *P. aeruginosa* [15], [23]–[25], [32]–[34]. Also the biosynthesis of the cytotoxic secondary metabolite pyocyanin is activated by the LasR/3OC_12_-HSL complex, though it starts later during the growth with respect to proteases and elastase biosynthesis [35]. Interestingly, when the wild type and *qteE* mutant cultures reached an A_600_≈3.5, the supernatants of the *qteE* mutant contained high pyocyanin levels, while this virulence factor was almost undetectable in the wild type strain (Fig. 3).

The growth curve of the *qteE* mutant was similar to those of the *rsaL* mutant and wild type strains and was not affected by the presence of the pBBR1MCS-5 vector, ruling out the possibility that differences in the growth rates could account for the diverse phenotypes described above (Fig. S1 in File S1). Moreover, complementation of the *qteE* mutant strain with the pQteE plasmid caused a repression of both 3OC_12_-HSL, proteases, elastase, and pyocyanin production, ruling out any involvement of possible artefacts caused by *qteE* mutagenesis on the tested phenotypes (Figs. 2 and 3). We also verified that the pBBR1MCS-5 empty vector did not affect the levels of 3OC_12_-HSL, proteases, elastase and pyocyanin either in the wild type or in the *qteE* mutant *per se* (Fig. S2 in File S1 and Fig. 3).

Overall, the above results are in agreement with previous studies showing that the *lasI* and *lasB* promoters are prematurely activated in the *qteE* mutant with respect to the wild type [17], [20], and that QteE overexpression causes repression of elastase and pyocyanin production in *P. aeruginosa* [20], [22]. Notably, our results demonstrate that in the *qteE* mutant the production of 3OC_12_-HSL and of main QS-controlled virulence factors is anticipated, and that these exo-products accumulate along growth at higher levels with respect to the wild type strain, supporting the hypothesis that *qteE* may play a role in *P. aeruginosa* pathogenesis.

### Effect of QS dysregulation on *P. aeruginosa* pathogenicity in a murine model of infection

A bacterial population can adopt distinct behaviours in acute and chronic infections, and distinctive phenotypes characterizing these two processes are often inversely regulated. An acute infection is rapid, systemic and carried out by a planktonic bacterial community expressing high levels of virulence factors. Conversely, in a chronic infection bacterial proliferation is limited to a specific host tissue (*e.g.*, in the CF lung or in association with medical devices), and bacteria can persist in the host for extended periods of time, adopting a slow-growing sessile lifestyle (biofilm). In the biofilm mode of growth bacteria are more resistant to the host immune system and prolonged antibiotic therapies, and they produce limited amount of virulence factors despite high cell density [29], [36].

In order to test the effect of pre-quorum and post-quorum dysregulation of the *lasI* gene *in vivo*, the virulence of *P. aeruginosa* PAO1 wild type and of its isogenic *qteE* and *rsaL* mutant strains was compared by using the “agar beads” murine model of lung infection [37]–[39]. In this model, *P. aeruginosa* cells embedded in agar beads are inoculated into the murine lungs, where replicate in microaerobic/anaerobic conditions in the form of microcolonies, similarly to the growth in the mucus of CF patients [40]. Mice dying within three days from the challenge are killed by a systemic (acute) infection. The surviving mice are sacrificed after 14 days from the challenge and bacterial load is evaluated in their lungs: the subset of survived mice containing *P. aeruginosa* (CFU ≥1000) in their lungs are considered chronically infected, while the others are considered cleared from the infection [37]–[39]. This murine model of infection has been previously used to test the virulence of a *P. aeruginosa* PAO1 QS mutant impaired in both 3OC_12_-HSL and C_4_-HSL production, showing that the QS mutant had strongly attenuated ability to cause chronic infection with respect to the wild type, while the two strains showed similar ability to kill mice within three days from the challenge [41]. In addition, this infection model has been used to validate the anti-virulence activity of anti-*Pseudomonas* compounds, including QS inhibitors [7], [42].

**Figure 4:**
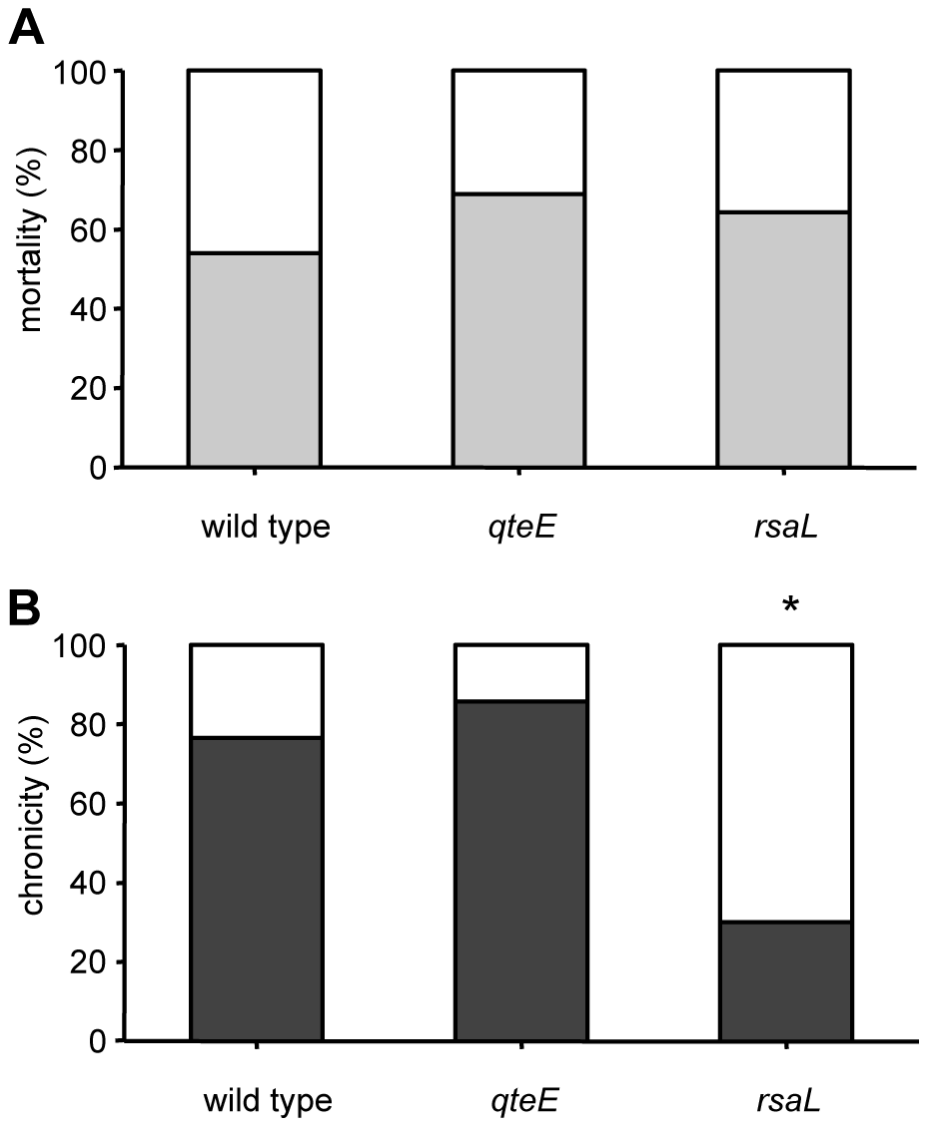
Effect of QS dysregulation caused by *qteE* and *rsaL* mutations on *P. aeruginosa* pathogenesis in mice. C57Bl/6 mice were infected with the indicated strains embedded in agar beads. (A) Mice mortality induced by bacteremia (light grey) and survival (white) were evaluated on challenged mice. (B) Clearance (white) and capacity to establish chronic airways infection (dark grey) were determined on surviving mice after 14 days from challenge. The results are averages of three independent experiments. Statistical significance is indicated by an asterisk comparing *P. aeruginosa* wild type versus *qteE* or *rsaL* strains (*p*<0.05).

When mice were challenged with the *P. aeruginosa* PAO1 wild type strain used in this study, 54% of mortality (20/37 mice) was observed in the first three days, while 76.5% of the survived mice (13/17 mice) were chronically infected after 14 days from the challenge (Fig. 4). Surprisingly, the ability of *qteE* mutant and of the wild type strains to cause both mice mortality and chronic infection, in the survived mice, did not show statistically significant differences (Fig. 4). Hence, despite the production of virulence factors regulated by QS is anticipated and increased in the *qteE* mutant *in vitro*, inactivation of this gene has no effect on *P. aeruginosa* ability to cause infection, at least in this model system.

Concerning the *rsaL* mutant, the ability of this strain to kill the mice by systemic infection (64.3%; 18/28 mice) did not show significant difference with respect to the wild type. This result was surprising, because the LD_50_ of the *rsaL* mutant is seven fold lower with respect to the wild type strain in the *G. mellonella* systemic infection model [26]. Although previous studies testing the virulence of QS defective or attenuated mutants showed a good correlation of the results between *G. mellonella* and murine infection models [43], it seems that this correlation is missing for mutants with dysregulated QS response.

Interestingly, only 30% (3/10 mice) of the survived mice developed a chronic infection after 14 days from the challenge with the *rsaL* strain, a percentage significantly lower than that observed with the wild type strain (76.5%; Fig. 4). Hence, the *rsaL* mutation decreased *P. aeruginosa* ability to cause chronic infection. This result strongly supports the hypothesis that the RsaL-mediated post-quorum homeostatic regulation of QS plays a positive role in the establishment of chronic lung infection in mice.

The surviving mice sacrificed 14 days from the challenge were either totally cleared (no *P. aeruginosa* cells in the lungs) or contained similar bacterial loads (>1000 CFU/lungs), independent from the *P. aeruginosa* strain used for the challenge (Fig. S3 in File S1). This result is overall in line with previous studies using this infection model [38].

## Conclusions

Since the discovery that *P. aeruginosa* virulence genes expression is QS-dependent and that QS mutants have attenuated pathogenicity in animal models of infection, researchers have tried to explain why QS favours the infection. An early and still in vogue theory is that the QS-control of virulence factors avoids the stimulation of the host immune response at early infection stages, when the size of the bacterial population is small [1]. Another hypothesis, not excluding the former one, is that QS could be important to save energy from unprofitable exoproducts production in environments with high mass transfer, allowing their synthesis only if bacteria are within a low diffusion rate environment, such as an infected tissue [44]. However, considering the key role played by the QS circuitry in *P. aeruginosa* central and secondary metabolism, and its poorly understood links with other cellular regulatory networks, it seems quite an hard task to find an univocal and simple explanation for the role played by QS in the infection.

*P. aeruginosa* has evolved as a tough versatile organism, able to thrive in a wide range of environmental niches rather than as a specialized pathogen. In accordance with its phenotypic plasticity, *P. aeruginosa* can cause a range of different acute and chronic infections in almost all areas of the human body [4], [16], [36], implying that the relevance of the different factors affecting the timing and extent of the QS response *in vivo* could be dependent upon the kind of infection.

In the lung infection model used in this study, the similar pathogenic behaviour of the *P. aeruginosa* wild type and *qteE* mutant indicates that the restrained production of 3OC_12_-HSL and expression of virulence genes in the pre-quorum period does not favour *P. aeruginosa* ability in either mice killing or in establishing a chronic lung infection. This finding argues against the hypothesis that delaying virulence factors production until cells amass to a certain density could favour the establishment of the infection [1], [20].

A relevant finding of this study is that the post-quorum homeostatic regulation of QS exerted by RsaL favours the establishment of the chronic *P. aeruginosa* infection. The importance of this factor also in the human chronic infection is supported by studies on *P. aeruginosa* strains isolated from the chronically infected lungs of CF patients. During the course of this infection, that can last for decades, the *P. aeruginosa* population that initially settles in the lung is subject to a microevolution process leading to the emergence of mutants with phenotypic traits unusual in the environmental strains, including loss of motility, increased ability to form biofilm, increased antibiotic resistance, reduced production of secreted virulence factors [45]. It is striking how these phenotypes inversely correlate with those disclosed by the *rsaL* mutant [26]. Accordingly, in the CF chronic lung infection there is a positive selection for cells expressing high levels of RsaL [46], [47].

Since *rsaL* transcription is strongly dependent upon LasR, this factor should not be expressed in a *lasR* mutant. Hence it is interesting to discuss our results by considering that *lasR* mutants are frequently isolated from the lungs of CF patients. It is still under debate whether these mutants arise because they are social cheaters gaining a growth advantage by utilizing “public goods” (*i.e.*, virulence factors) produced by neighbour wild type cells, rather than producing their own [45], [48], [49], or whether they are better adapted than the wild type to the peculiar environment of the CF lung [50], [51]. Overall, it is still unclear whether and how the emergence of *lasR* mutants could contribute to the CF lung decline. However, a recent work showed that *lasR* mutants were able to produce very high levels of pyocyanin under the slow-growing conditions typical of the chronic infection, while wild type cells did not [13]. Moreover, in co-cultivation experiments, the *lasR* mutant was able to cooperate with the wild type for pyocyanin production [13]. Pyocyanin overproduction in the *lasR* mutant is due to the loss of repression normally exerted by RsaL on phenazine biosynthetic genes, because RsaL itself is not expressed as a consequence of *lasR* mutation [13]. However, mutations in *rsaL* are not commonly isolated in CF clinical samples, suggesting that the constitutive expression of QS regulated factors caused by this mutation is unfavourable in the CF lung environment, and that a mutation in the *rsaL* gene can be tolerated only when associated to the lack of expression of the entire LasR regulon.

It has been proposed that targeting the function and the cellular levels of the regulatory factors that modulate the QS pre-quorum and post-quorum response could be a strategy to inhibit *P. aeruginosa* virulence [31]. Though the hypervirulent phenotype disclosed by the *rsaL* mutant *in vitro* and in the *G. mellonella* infection model might cause some concern, our results indicate that a compound targeting RsaL could reduce the ability of *P. aeruginosa* to establish a chronic infection. Moreover, since the *rsaL* mutant is also less resistant to antibiotics, with respect to the wild type [26], such compound could synergize with drugs currently used in the CF therapy.

In conclusion, our results contribute to a better understanding of the QS regulatory factors involved in the establishment of the chronic infection caused by *P. aeruginosa*, indicate the RsaL homeostatic regulator of QS as a promising target for drugs specific against this kind of infection, and highlight the importance of carrying out further studies about the role played by QS modulation in mammalian infection models.

## Materials and Methods

### Bacterial strains and culture conditions

*Pseudomonas aeruginosa* wild type, substrain PAO1-UW, and its *qteE* and *rsaL* mutant derivatives were supplied by The University of Washington Genome Center (www.genome.washington.edu/UWGC/pseudomonas) [52]. *Escherichia coli* DH5α [53] was used for cloning purposes. Bacterial strains were grown at 37°C in Luria-Bertani broth (LB) [54] with 200 r.p.m shaking; 20 mg/L and 100 mg/L Gentamicin was added to the *E. coli* and *P. aeruginosa* strains for plasmid maintenance, respectively.

### Plasmids construction

A DNA region of 1,280 bp encompassing the entire *qteE* gene, including its native promoter region, was amplified from the PAO1-UW genome by PCR and cloned into the KpnI-HindIII sites of the pBBR1MCS-5 vector [55], generating the pQteE plasmid. The PCR was performed with the following Forward and Reverse oligonucleotides: 5′-CGGGGTACCGAGGACTACCAGAAAGCCC-3′, and 5′-ATAAAGCTTTCAGGCCAGCCCATAGCT-3′; the KpnI and HindIII restriction sites introduced in the oligonucleotides are underlined.

### Phenotypic assays

Plasmids pBBR1MCS-5 and pQteE were inserted in the PAO1-UW strain and in the PAO1-UW *qteE* mutant by conjugation, as previously described [56]. Strains were grown 16 hours at 37°C in LB supplemented with 100 µg/ml Gentamicin. For the phenotypic assays, cultures were diluted to an A_600_ of 0.02 in LB and incubated at 37°C with 200 r.p.m shaking. Cell-free supernatants were collected every hour after 3 hours of incubation. The concentration of 3OC_12_-HSL, proteases, elastase and pyocyanin in the cell-free supernatants were measured as previously described [24], [57]–[60].

The average measurements and relative standard deviations were calculated from three independent experiments.

### Mouse model of *P. aeruginosa* lung infection

C57Bl/6 male mice (20–22 gr) were purchased by Charles River Laboratories (Calco, Italy). All mice were maintained under specific pathogen-free conditions in sterile cages which were put into a ventilated isolator. Fluorescent lights were cycled 12 hours on/12 hours off, and ambient temperature (23±1°C) and relative humidity (40–60%) were regulated. The *P. aeruginosa* agar-beads mouse model was used [37]. PAO1-UW wild type strain and the isogenic *qteE* and *rsaL* mutant strains were used for inclusion in the agar beads, as previously described [38], [39], [61]. Briefly, mice were anesthetized with 2.5% avertin (2,2,2-tribromethanol, 97%; Sigma Aldrich) in 0.9% NaCl, intubated with a 22-gauge venous catheter and inoculated with *P. aeruginosa* 2×10^6^ CFU. Animals were observed twice a day and those showing more than 25% of body weight loss and had evidence of severe clinical disease, such as scruffy coat, inactivity, loss of appetite, poor locomotion, or painful posture, were sacrificed before the termination of the experiments with an overdose of carbon dioxide. Fourteen days after infection, mice were sacrificed by CO_2_ administration and murine lungs were excised, homogenized and plated onto Trypticase Soy Agar plates for CFU counting. Recovery of ≥1,000 CFU from lung cultures was indicative of chronic infection [37], [38]. The results are averages of at least three independent experiments. Overall, 37, 45 and 28 mice were infected with the *P. aeruginosa* wild type, the *qteE* mutant or the *rsaL* mutant strains, respectively (10-12 mice for each experimental group, for experiment).

Statistical analysis was performed using Fisher's exact test (two-tailed) for categorical variables. Differences were considered statistically significant at *p* value <0.05.

### Ethics statement

Animal studies were carried out according to protocols approved by the IRCCS - San Raffaele Scientific Institute (Milan, Italy) Institutional Animal Care and Use Committee (IACUC), and in strict accordance with the Italian Ministry of Health guidelines for the use and care of experimental animals.

## Supporting Information

File S1 **This file contains Figures S1-S3.** Figure S1, Growth curve of *P. aeruginosa* PAO1 wild type, *qteE* and *rsaL* strains. Figure S2, The presence of pBBR1MCS-5 vector does not affect virulence-related phenotypes in *P. aeruginosa*. Figure S3, Bacterial load in the lungs of mice infected with *P. aeruginosa*.
(PDF)
