# Supplementary material for: Affecting Pseudomonas aeruginosa Phenotypic Plasticity by Quorum Sensing Dysregulation Hampers Pathogenicity in Murine Chronic Lung Infection
Source: PLoS One. 2014 Nov 24;9(11):e112105. doi: 10.1371/journal.pone.0112105 (PMC4242533; doi:10.1371/journal.pone.0112105)
Supplement: File S1 — This file contains Figures S1-S3. Figure S1, Growth curve of P. aeruginosa PAO1 wild type, qteE and rsaL strains. Figure S2, The presence of pBBR1MCS-5 vector does not affect virulence-related phenotypes in P. aeruginosa. Figure S3, Bacterial load in the lungs of mice infected with P. aeruginosa. (PDF) [file pone.0112105.s001.pdf]

Affecting *Pseudomonas aeruginosa* phenotypic plasticity by quorum sensing dysregulation hampers pathogenicity in murine chronic lung infection

Roslen Bondí, Marco Messina, Ida De Fino, Alessandra Bragonzi, Giordano Rampioni, Livia Leoni

SUPPORTING INFORMATION

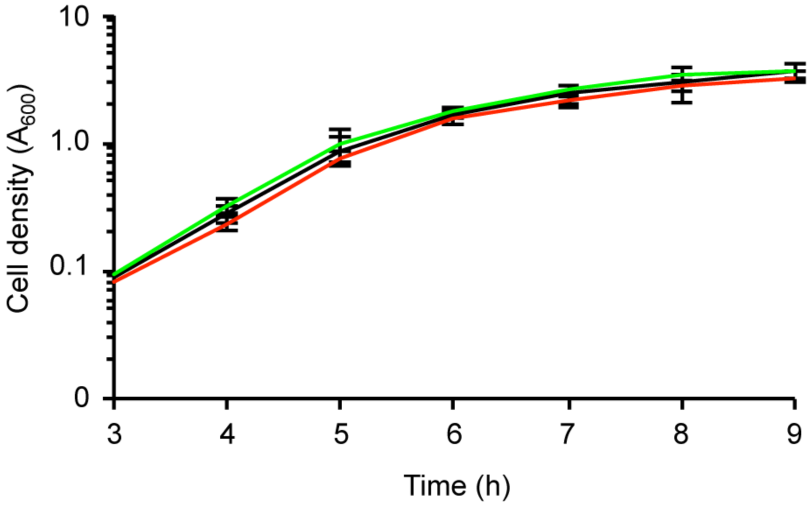

Figure S1. Growth curve of *P. aeruginosa* PAO1 wild type, *qteE* and *rsaL* strains.
Cell density measured in *P. aeruginosa* PAO1 wild type (black line), *qteE* (red line) and *rsaL* strains (green line). Values are the means (± standard deviations) of at least three independent experiments. The pBBR1MCS-5 or pQteE (pBBR1MCS-5-derived) plasmids did not affect the growth curves (data not shown).

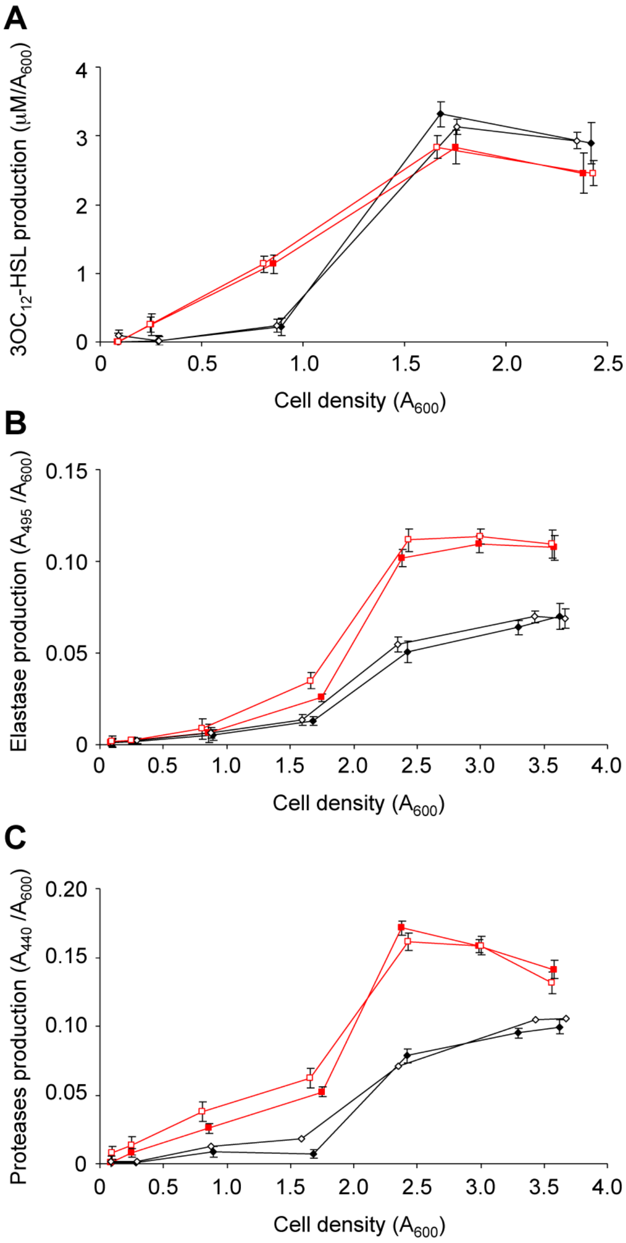

Figure S2. The presence of pBBR1MCS-5 vector does not affect virulence-related phenotypes in *P. aeruginosa.*

Levels of (A) $3OC_{12}$-HSL, (B) elastase, (C) proteases produced along growth by *P. aeruginosa* wild type (black lines) and *qteE* (red lines) strains containing or not the pBBR1MCS-5 empty vector (full and open circles, respectively). Values are the means (± standard deviations) of at least three independent experiments.

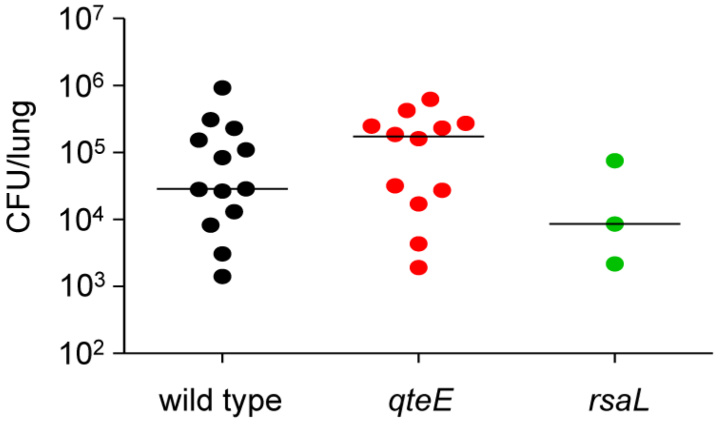

Figure S3. Bacterial load in the lungs of mice infected with *P. aeruginosa.*
Colony-forming units (CFU) per lung were counted after 14 days from challenge. Black full circles, wild type; red full circles, *qteE* mutant; green full circles, *rsaL* mutant. No statistically significant differences between groups were observed ($0.19 \leq p \leq 0.67$; unpaired two-tailed *t* test).
